# Sociodemographic correlates of prospective dog owners’ intentions to participate in controlled trials of dog ownership and human health

**DOI:** 10.1186/s13104-018-3277-x

**Published:** 2018-03-12

**Authors:** Debbie Chia, Lauren Powell, Vanessa Lee, Marjan Mosalman Haghighi, Anthony Podberscek, Ding Ding, Cathie Sherrington, Emmanuel Stamatakis

**Affiliations:** 1 Prevention Research Collaboration, Sydney School of Public Health, The University of Sydney, Sydney, 2006 Australia; 2 Faculty of Health Sciences, The University of Sydney, Sydney, 2006 Australia; 3 Sydney School of Veterinary Science, The University of Sydney, Sydney, 2006 Australia; 4 Musculoskeletal Health Sydney, Sydney School of Public Health, The University of Sydney, Sydney, 2006 Australia

**Keywords:** Dog ownership, Public health, Research participation, Participation incentives

## Abstract

**Objective:**

Dog ownership is popular, with research suggesting improvements in physical and psychological health of dog owners. However, majority of these studies were not investigator-controlled. Ethical and practical implications arising from the intervention exposure (dog ownership) result in recruitment difficulties. A fit-for-purpose design, such as delaying dog adoption until after data collection, could alleviate such issues. The purpose of this study was to explore intentions and possible incentives for participation in investigator-controlled trials examining the effects of dog ownership on human physical and psychological health.

**Results:**

Female (OR 1.64, 95% CI 1.31–2.04) and older (OR 65+ years 1.49, 95% CI 1.06–2.10) participants were more likely to be interested in taking part in a study investigating the health benefits of dog ownership. Majority reported no incentive was necessary for participation (57%), while others preferred pet food supplies (37%), or vouchers for veterinary care (32%). Over half of participants (53%) were willing postpone adoption for up to 3 months to participate in an investigator-controlled trial. The results of the study, showing majority of participants interested in participating in future studies examining the health benefits of dog ownership and without incentives, provides insight to methodical directions for future studies.

**Electronic supplementary material:**

The online version of this article (10.1186/s13104-018-3277-x) contains supplementary material, which is available to authorized users.

## Introduction

Dog ownership is common around the world; for example, approximately 50% of United States, 39% of Australian, and 27% of UK households own a dog [1]. Some research indicates potential health benefits resulting of dog ownership, including increased physical activity in dog owners compared with non-owners [2–7], enhanced social support [5, 8], and improvements in mental health [9, 10]. However, the majority of these studies relied on self-reported measures of human health [2–4, 6–8, 10]. In the absence of robust scientific evidence, promoting and enabling dog ownership as a means of tackling physical inactivity, cardiovascular disease, and mental illness is difficult. Due to ethical and practical complications of examining dog ownership and its effect on human health in an investigator-controlled setting, existing studies are largely cross-sectional or retrospective. Conducting a classic randomised controlled trial (RCT), in which groups of participants are allocated to owning a dog, is problematic; such a design would raise potentially irreconcilable animal and human welfare issues. A potential fit-for-purpose study design could consist of two groups who intend to acquire a dog; one who will acquire a dog imminently and the other is willing to postpone acquisition until after the required data collection period. This will allow allocation of participants into two groups (imminent and delayed adoption), while ensuring ethical standards are upheld. Given that such trials have never been conducted, it is not known whether participants would willingly postpone acquisition of a dog for the purpose of research. Moreover, there is a lack of data on what type of incentives should be supplied to attain the best possible level of recruitment of participants.

The purpose of the current study was to (a) examine the characteristics of people willing to participate in prospective controlled trials investigating the effects of dog ownership on human physical and psychological health; (b) determine which incentives will encourage participation in prospective controlled trials in this field of research, and; (c) determine which incentives will encourage participation with an adoption waiting list of up to 3 months. It is expected that participants will be less likely to participate if they are expected to wait up to 3 months for adoption, or would require additional incentives. Results from this study will contribute to the development of future community-based controlled trials on dog ownership and human health.

## Main text

### Methods

A convenience sampling design was used. Participants were recruited online from May 4th to October 25th 2016 through Pet Rescue (https://www.petrescue.com.au), an Australia-wide animal welfare charity with an online directory of homeless pets and pet adoption organisations [11]. Visitors to the website who clicked on ‘search for a dog’ were presented with a pop-up screen asking if they were willing to take part in an online survey. After providing informed consent, the individual was invited to complete the survey. Power and sample size calculations were not conducted; the sample size attained was sufficient and comparable to other lifestyle-related online surveys [12, 13].

#### Questionnaire

Data was retrieved from specific questions and forms part of a larger questionnaire. These questions were:

1. If you were asked to take part in a study investigating physical and mental health benefits of dog ownership, would you be interested in taking part?
2. Which of the following incentives would you prefer to receive if you participated in a study?
3. If you were offered material incentives, would you still be interested in participating if you were asked to go on a waiting list for up to 3 months before you adopt a dog? *This* 3-month *waiting period would ensure that the study is of the best possible quality.*
4. What incentive would you need to receive to guarantee that you would adopt if you were put on a waiting list for up to 3 months?

The full 25-item questionnaire also addressed reasons for considering dog adoption; perceived physical, mental, and social health benefits from owning a dog, and possible challenges of dog ownership. Information on participants’ demographic characteristics and previous dog ownership status were also obtained. Previous dog ownership status refers to whether the participant had previously owned a dog within the last 10 years. Questions not analysed in this study will be reported elsewhere.

#### Data analysis

The first part of the analysis examined any intentions to take part in a study investigating the physical and psychological health benefits of dog ownership and the second analysis examined any intentions for participants to take part in a study with a waiting list of up to 3 months. All statistical analyses were performed with SPSS software (version 22.0; SPSS Inc, Chicago, IL).

Multiple logistic regression was used to examine the associations between sociodemographic characteristics (gender, age, education, and dog ownership history) and intentions to take part in a future trial that will involve postponing a planned dog adoption for up to 3 months. Model 1 was unadjusted while Model 2 was adjusted for age, gender, education, and dog ownership status. Cross-tabulations examined the associations between the sociodemographic characteristics of gender and age with the top five incentives necessary to take part in further research studies on dog ownership and human health. Statistical significance was defined as a two tailed p ≤ 0.05.

Participants from the online survey were included in the analyses if they completed at least 70% or more of the survey and reported a plausible age value (< 103 years, age of the oldest participant in the New South Wales–based 45 and Up study [14]). The 70% completion criterion was set to ensure that the analytical sample had mostly completed data for each participant.

### Results

The full dataset included 3920 participants. Of these, 449 participants were excluded for not completing at least 70% of the survey or not providing details of their age and gender. Another seven participants were excluded due to implausible age values. The final analysis included a sample of 3472 participants (Table 1).

**Table 1:** Demographic characteristics of total survey sample (n = 3472)

Characteristic	n	%
Gender		
Male	529	15.2
Female	2942	84.8
Age (years)		
18–44	1815	52.3
45–64	1352	39.0
65+	304	8.8
Education		
Less than year 12 or equivalent	276	8.0
Year 12 or equivalent	640	18.6
Trade certificate/diploma	616	17.9
Bachelor’s degree/postgrad	1913	55.5
Dog ownership status		
Previous dog owner	1903	54.8
Current dog owner	1362	39.2
Non-dog owner	200	5.8

Total number of participants may differ due to missing data

*N* number of participants in sample, *Total %* total percentage of sample

Female (69.4%), younger (18–44 years; 41.1%), and university educated (45.1%) participants were more likely to intend to take part in a study on dog ownership and human health with an adoption waiting list of up to 3 months (Table 2). Nearly half (44%) of participants had previously owned a dog and 32.2% currently owned a dog.

**Table 2:** Participant characteristics by intentions to participate with and without a dog adoption waiting list of up to 3 months

Variable	Cases (n = 3472)	Yes (%)	No (%)
Gender	3328		
Male		373 (11.2)	132 (4.0)
Female		2309 (69.4)	514 (15.4)
Age (years)	3328		
18–44		1369 (41.1)	373 (11.2)
45–64		1073 (32.2)	225 (6.8)
65+		240 (7.2)	48 (1.4)
Education	3305		
Less than year 12 or equivalent		208 (6.3)	54 (1.6)
Year 12 or equivalent		491 (14.9)	122 (3.7)
Trade certificate/diploma		474 (14.9)	109 (3.3)
Bachelor’s degree/postgrad		1492 (45.1)	355 (10.7)
Dog ownership status	3322		
Previous dog owner		1463 (44.0)	360 (10.8)
Current dog owner		1071 (32.2)	243 (7.3)
Non-dog owner		142 (4.3)	43 (1.3)
Gender	2492		
Male		247 (9.9)	109 (4.4)
Female		1575 (63.2)	561 (22.5)
Age (years)	2492		
18–44		943 (37.8)	330 (13.2)
45–64		729 (29.3)	269 (10.8)
65+		150 (6.0)	71 (2.8)
Education	2477		
Less than year 12 or equivalent		144 (5.8)	51 (2.1)
Year 12 or equivalent		339 (13.7)	115 (4.6)
Trade certificate/diploma		331 (13.4)	114 (4.6)
Bachelor’s degree/postgrad		999 (40.3)	384 (15.5)
Dog ownership status	2486		
Previous dog owner		1003 (28.3)	355 (11.5)
Current dog owner		704 (40.3)	287 (14.3)
Non-dog owner		109 (4.4)	28 (1.1)

As there were no major differences between the results of the unadjusted Model 1 and the adjusted Model 2 the text below refers to the fully-adjusted results (Model 2) unless otherwise stated.

#### Intentions to take part in further research

Table 3 describes the associations between gender, age, education level, and dog ownership status and intentions to take part in a research study. Most participants (n = 2682, 77.3%) indicated they were interested in taking part in a study investigating the physical and mental health benefits of dog ownership. Women were more likely than men to indicate an interest (OR females 1.64, CI 1.31–2.04). Older participants had higher odds of indicating an interest in participating (OR 65+ years age 1.49, CI 1.06–2.10).

**Table 3:** Multiple logistic regression describing the associations between sociodemographic variables and intentions to participate

		Model 1	Model 2
Variable	Cases (n = 3472)	OR (95% CI)	OR (95% CI)
Any interest to take part in a study on dog ownership and human health			
Dog ownership status	3299		
Non-dog owner		1	1
Previous dog owner		1.24 (.86–1.77)	1.17 (.81–1.68)
Current dog owner		1.36 (.94–1.97)	1.25 (.86–1.82)
p value		.215	.456
Gender	3300		
Male		1	1
Female		1.58 (1.27–1.98)	1.64 (1.31–2.04)
p value		< .001	< .001
Age (years)	3300		
18–44		1	1
45–64		1.30 (1.08–1.56)	1.31 (1.–1.58)
65+		1.38 (.986–1.93)	1.49 (1.06–2.10)
p value		<.01	<.01
Education	3300		
Less than year 12 or equivalent		1	1
Year 12 or equivalent		1.04 (.73–1.49)	1.12 (.78–1.62)
Trade certificate/diploma		1.12 (.78–1.62)	1.21 (.84–1.75)
Bachelor’s degree/postgrad		1.09 (.79–1.50)	1.17 (.84–1.62)
p value		.910	.759
Any interest to take part in a study with a waiting list of up to 3 months			
Dog ownership status	2471		
Non-dog owner		1	1
Previous dog owner		.74 (.48–1.13)	.76 (.49–1.17)
Current dog owner		.64 (.41–.99)	.64 (.41–1.00)
p value		.079	.066
Gender	2472		
Male		1	1
Female		1.23 (.96–1.57)	1.22 (.95–1.56)
p value		.106	.126
Age (years)	2472		
18–44		1	1
45–64		.95 (.79–1.15)	.96 (.79–1.16)
65+		.75 (.55–1.03)	.77 (.56–1.07)
p value		.206	.292
Education	2472		
Less than year 12 or equivalent		1	1
Year 12 or equivalent		1.04 (.71–1.53)	1.00 (.68–1.48)
Trade certificate/diploma		1.02 (.70–1.50)	1.01 (.69–1.49)
Bachelor’s degree/postgrad		.92 (.65–1.29)	.88 (.62–1.25)
p value		.690	.554

Model 1 unadjusted

Model 2 is adjusted for age, sex, education level and dog ownership status

*OR* odd ratio, *CI* confidence interval, *N* number of participants in sample

Over half (52.5%) of participants indicated they would still be interested in participating even if they were asked to go on a waiting list for up to 3 months prior to adoption. The willingness to participate despite the wait did not differ statistically by gender, age, education level, and dog ownership status.

#### Incentives to take part in further research

Participants who indicated they were interested in participating in a study examining the effects of dog ownership on human physical and mental health were asked to select their most preferred incentives. These were pet food supplies (36.8%), vouchers for vet care (32.3%), refund of the adoption fee (23.6%), and a free veterinary appointment (22.4%) (Additional file 1: Table S1). Among the 56.7% of participants who stated no incentive was necessary to take part in future studies, 48.5% were female and 26.9% were 18–44 years old.

The preferred incentives for participation in a study with a waiting list of up to 3 months were a refund of adoption fees (27.2%), vouchers for vet care (26.4%), free veterinary appointments (19.7%), and free pet food for a few months (17.7%) (Additional file 1: Table S1). Nearly half (45.7%) did not require an incentive to participate in a study with a waiting list of up to 3 months.

### Discussion

Conducting community controlled trials that examine the influence of real-world dog ownership on human health is particularly challenging and recruitment strategies need to be carefully developed and thoroughly tested. This study takes the first steps towards understanding the intentions of individuals to participate in controlled trials investigating the effects of dog ownership on human health, and the incentives preferred to motivate participation. Knowledge of the attitudes of potential research participants is necessary to overcome current methodological limitations and facilitate the development of robust future controlled trials.

This study showed that women were more likely to indicate an interest in participating in future prospective studies investigating the impact of dog ownership and human health. This aligns with previous studies that report women were more likely to be the primary carer of a dog than men [11–13]. The study also identified a statistically significant association between age and intention to participate; older respondents more commonly indicated an interest in research participation than younger respondents. This contrasts current reports of the demographics of dog owners, which show that individuals under the age of 44 are more likely to own a dog than older individuals [15]. Similarly, households with older children and young adults are also more likely to own a dog than those without [16]. This suggests that older individuals, who are less likely to currently own a dog, show greater interest in participation and may represent a target group with potentially high participation rates. These findings also indicate that recruiting male and younger participants will pose a challenge when conducting future trials and more efforts are required to ensure the sample is demographically representative of the population.

Our findings show that the majority of participants would not require any incentives to take part in future research. When considering a study involving a 3-month waiting list, nearly half of participants still indicated that no incentive was necessary to encourage participation. Incentives have been suggested to reduce the scientific integrity of studies by introducing the risk of coercion and sample bias [17, 18]. It is therefore of significant value to conduct research without the use of incentives, if participation rates are feasible.

#### Implications and conclusions

This study assists future researchers when planning community-based trials to investigate the influence of dog ownership on human health by indicating which groups are best targeted for recruitment. Incentives may not always be required for future studies as our results indicate that research in this field is feasible without them. If necessary, pet food supplies, vouchers for veterinary care, and a refund of the adoption fee could be the most effective incentives in motivating participation. This study provides methodological direction for future trials in this area of research and may increase the potential for a robust study design with high recruitment rates.

## Limitations

Strengths of the current study include the nation-wide coverage and large sample size. There are a number of important limitations of this study. Firstly, there is a lack of information about non-respondents and limited information about respondents who did not meet the 70% survey completion criteria; missing demographic data was the main reason of not meeting the criteria. Demographic similarities and differences between respondents and non-respondents therefore cannot be determined. Convenience sampling is another limitation; the current study only considers respondents who were actively seeking a dog for adoption through the Pet Rescue website. People who are looking for a rescue dog through other online (e.g. independent websites, social media) and non-online sources are therefore not captured in this survey. However, Pet Rescue is the largest online animal adoption organisation in Australia consisting of small independent rescue groups, animal rescue shelters, veterinary practices and council-operated pound facilities. People looking to acquire a dog through breeders and pet stores are also not represented in the current study. Lastly, although potential participants indicate they will participate in such a study, their actions may not reflect their intentions. This needs to be tested in future studies.

## Additional file

**Additional file 1: Table S1.** Sociodemographic characteristics of the top five incentives necessary to take part in further research studies on dog ownership and human health. It describes the top five preferred incentives for participation in a study examining the physical and mental health benefits of dog ownership on human health with and without a waiting list of up to 3 months

## Abbreviations

CI: confidence intervals
OR: odd’s ratio
RCT: randomized controlled trial
