# Supplementary material for: Sociodemographic correlates of prospective dog owners’ intentions to participate in controlled trials of dog ownership and human health
Source: BMC Res Notes. 2018 Mar 12;11:169. doi: 10.1186/s13104-018-3277-x (PMC5848557; doi:10.1186/s13104-018-3277-x)
Supplement: Supplementary file 1 — Additional file 1: Table S1. Sociodemographic characteristics of the top five incentives necessary to take part in further research studies on dog ownership and human health. It describes the top five preferred incentives for participation in a study examining the physical and mental health benefits of dog ownership on human health with and without a waiting list of up to 3 months [file 13104_2018_3277_MOESM1_ESM.docx]

**Table S1:** Sociodemographic characteristics of the top five incentives necessary to take part in further research studies on dog ownership and human health

| Variable | Cases (n=3471) | Yes % | No % |
| --- | --- | --- | --- |
| *Preferred incentives for participation in a study on the physical and mental health benefits of dog ownership on human health* | | | |
|  |  |  |  |
| *Pet food supplies* | 2682 |  |  |
| Gender |  |  |  |
| Male |  | 4.8 | 9.1 |
| Female |  | 32 | 54.1 |
| *Total* |  | 36.8 | 63.2 |
|  |  |  |  |
| Age (Years) |  |  |  |
| 18-44 |  | 24.4 | 26.7 |
| 45-64 |  | 10.7 | 29.3 |
| 65+ |  | 1.7 | 7.2 |
| *Total* |  | *36.8* | *63.2* |
|  |  |  |  |
| *Vouchers for vet care* | 2682 |  |  |
| Gender |  |  |  |
| Male |  | 4.1 | 9.8 |
| Female |  | 28.2 | 57.9 |
| *Total* |  | 32.3 | 67.7 |
|  |  |  |  |
| Age (Years) |  |  |  |
| 18-44 |  | 18.6 | 32.4 |
| 45-64 |  | 11.6 | 28.4 |
| 65+ |  | 2.1 | 6.9 |
| *Total* |  | 32.3 | 67.7 |
|  |  |  |  |
| *Refund of adoption fee* | 2682 |  |  |
| Gender |  |  |  |
| Male |  | 3.1 | 10.8 |
| Female |  | 20.5 | 65.6 |
| *Total* |  | 23.6 | 76.4 |
|  |  |  |  |
| Age (Years) |  |  |  |
| 18-44 |  | 14.4 | 36.7 |
| 45-64 |  | 7.4 | 32.6 |
| 65+ |  | 1.8 | 7.1 |
| *Total* |  | 23.6 | 76.4 |
|  |  |  |  |
| *Free veterinary appointment* | 2682 |  |  |
| Gender |  |  |  |
| Male |  | 3.2 | 10.7 |
| Female |  | 19.2 | 66.9 |
| *Total* |  | 22.4 | 77.6 |
|  |  |  |  |
| Age (Years) |  |  |  |
| 18-44 |  | 14.3 | 36.8 |
| 45-64 |  | 6.7 | 33.3 |
| 65+ |  | 1.5 | 7.5 |
| *Total* |  | 22.4 | 77.6 |
|  |  |  |  |
| *No incentive is necessary* | 2682 |  |  |
| Gender |  |  |  |
| Male |  | 8.2 | 5.7 |
| Female |  | 48.5 | 37.6 |
| *Total* |  | 56.7 | 43.3 |
|  |  |  |  |
| Age (Years) |  |  |  |
| 18-44 |  | 26.9 | 24.2 |
| 45-64 |  | 24.2 | 15.8 |
| 65+ |  | 5.6 | 3.3 |
| *Total* |  | 56.7 | 43.3 |
|  |  |  |  |
| *Preferred incentives for participation in a study with a waiting list of up to 3 months* | | | |
|  |  |  |  |
| *Free pet food for a few months* | 1822 |  |  |
| Gender |  |  |  |
| Male |  | 2.9 | 10.6 |
| Female |  | 14.8 | 71.7 |
| *Total* |  | 17.7 | 82.3 |
|  |  |  |  |
| Age (Years) |  |  |  |
| 18-44 |  | 13.6 | 38.1 |
| 45-64 |  | 3.7 | 36.3 |
| 65+ |  | 0.3 | 7.9 |
| *Total* |  | 17.7 | 82.3 |
|  |  |  |  |
| *Vouchers for vet care* | 1822 |  |  |
| Gender |  |  |  |
| Male |  | 3.8 | 9.7 |
| Female |  | 22.6 | 63.9 |
| *Total* |  | 26.4 | 73.6 |
|  |  |  |  |
| Age (Years) |  |  |  |
| 18-44 |  | 16.1 | 35.6 |
| 45-64 |  | 8.9 | 31.1 |
| 65+ |  | 1.3 | 6.9 |
| *Total* |  | 26.4 | 73.6 |
|  |  |  |  |
| *Refund of adoption fee* | 1822 |  |  |
| Gender |  |  |  |
| Male |  | 3.6 | 10.0 |
| Female |  | 23.7 | 62.8 |
| *Total* |  | 27.2 | 72.8 |
|  |  |  |  |
| Age (Years) |  |  |  |
| 18-44 |  | 17.1 | 34.7 |
| 45-64 |  | 8.6 | 31.4 |
| 65+ |  | 1.5 | 6.7 |
| *Total* |  | 27.2 | 72.8 |
|  |  |  |  |
| *Free veterinary appointment* | 1822 |  |  |
| Gender |  |  |  |
| Male |  | 2.4 | 11.1 |
| Female |  | 17.3 | 69.2 |
| *Total* |  | 19.7 | 80.3 |
|  |  |  |  |
| Age (Years) |  |  |  |
| 18-44 |  | 12.7 | 39.0 |
| 45-64 |  | 5.7 | 34.4 |
| 65+ |  | 1.3 | 6.9 |
| *Total* |  | 19.7 | 80.3 |
|  |  |  |  |
| *No incentive is necessary* | 1822 |  |  |
| Gender |  |  |  |
| Male |  | 6.6 | 7.0 |
| Female |  | 39.1 | 47.4 |
| *Total* |  | 45.7 | 54.3 |
|  |  |  |  |
| Age (Years) |  |  |  |
| 18-44 |  | 19.8 | 32.0 |
| 45-64 |  | 20.9 | 19.2 |
| 65+ |  | 5.0 | 3.2 |
| *Total* |  | 45.7 | 54.3 |
|  |  |  |  |
| N; Number of Participants in sample, Yes %; Total percentage of participants in sample that responded Yes, No %; Total percentage of participants in sample that responded No, Total; Total percentage of sample | | | |
